# Predicting the survival of patients with pancreatic neuroendocrine neoplasms using deep learning: A study based on Surveillance, Epidemiology, and End Results database

**DOI:** 10.1002/cam4.5949

**Published:** 2023-05-11

**Authors:** Chen Jiang, Kan Wang, Lizhao Yan, Hailing Yao, Huiying Shi, Rong Lin

**Affiliations:** ^1^ Department of Gastroenterology, Union Hospital, Tongji Medical College Huazhong University of Science and Technology Wuhan China; ^2^ Department of Cardiovascular Surgery, Union Hospital, Tongji Medical College Huazhong University of Science and Technology Wuhan China; ^3^ Department of Hand Surgery, Union Hospital, Tongji Medical College Huazhong University of Science and Technology Wuhan China

**Keywords:** deep learning, DeepSurv, pancreatic neuroendocrine neoplasms, survival analysis

## Abstract

**Background:**

The study aims to evaluate the performance of three advanced machine learning algorithms and a traditional Cox proportional hazard (CoxPH) model in predicting the overall survival (OS) of patients with pancreatic neuroendocrine neoplasms (PNENs).

**Method:**

The clinicopathological dataset obtained from the Surveillance, Epidemiology, and End Results database was randomly assigned to the training set and testing set at a ratio of 7:3. The concordance index (*C*‐index) and integrated Brier score (IBS) were used to compare the predictive performance of the models. The accuracy of the model in predicting the 5‐year and 10‐year survival rates was compared using the receiver operating characteristic curve, decision curve analysis (DCA) and calibration curve.

**Results:**

This study included 3239 patients with PNENs in total. The DeepSurv model had the highest *C*‐index of 0.7882 in the testing set and training set and the lowest IBS of 0.1278 in the testing set compared with the CoxPH, neural multitask logistic and random survival forest models (*C*‐index = 0.7501, 0.7616, and 0.7612, respectively; IBS = 0.1397, 0.1418, and 0.1432, respectively). Moreover, the DeepSurv model had the highest accuracy in predicting 5‐ and 10‐year OS rates (area under the curve: 0.87 and 0.90). DCA showed that the DeepSurv model had high potential for clinical decisions in 5‐ and 10‐year OS models. Finally, we developed an online application based on the DeepSurv model for clinical use (https://whuh‐ml‐neuroendocrinetumor‐app‐predict‐oyw5km.streamlit.app/).

**Conclusions:**

All four models analyzed above can predict the prognosis of PNENs well, among which the DeepSurv model has the best prediction performance.

## 1 INTRODUCTION

Neuroendocrine tumors (NENs) originate from neuroendocrine cells and can occur in all organs; the most common sites are the gastrointestinal tract, pancreas, and lungs.^1^ Although rare, the incidence of NENs has increased significantly over the past decades, reaching 6.98 cases per 100,000 people as reported by the Surveillance, Epidemiology, and End Results (SEER) program.^2^ As a subgroup of NENs, pancreatic neuroendocrine neoplasms (PNENs) account for approximately 7% of all NENs, and the highest incidence reaches 0.48 per 10,0000 people.^2^, ^3^

PNENs are classified as nonfunctional and functional tumors according to whether they have hormone secretion function and hormone‐induced clinical symptoms. Nonfunctional PNENs account for 60%–90% of PNENs, and functional PNENs are rare and mainly include insulinomas, glucagonomas, gastrinomas, growth hormone tumors, vasoactive intestinal polypeptide (VIP) tumors, and adrenocorticotropic hormone tumors.^4^ Due to the highly heterogeneous biological behavior and complex clinical manifestations, there is a great challenge for physicians to make early diagnosis and prognosis evaluations. According to the SEER database, the median survival time of PNEN patients with distant metastasis is only 27 months, and the 5‐year and 10‐year overall survival (OS) rates are only 27% and 11%, respectively.^5^ Even after radical surgery, the 5‐year recurrence and metastasis rates range from 21% to 47%.^6^, ^7^ Consequently, accurate models are necessary to predict the prognosis of PNENs.

TNM stage, tumor size, organ invasion, and Ki‐67 index have been included in the prognostic system by the European Neuroendocrine Tumor Society (ENETS) and American Joint Committee on Cancer (AJCC), but factors such as age, sex, tumor number or therapy methods are still lacking.^8^, ^9^ Therefore, a comprehensive accurate survival prediction model made up of numerous clinicopathological features is necessary to contribute to treatment decisions and disease surveillance.

With the continuous development of computer technology, machine learning (ML) technology has provided a new method for tumor diagnosis and management.^10^ It can use information from large datasets to create more accurate prediction models. Therefore, we aim to assess the performance of three advanced ML algorithms and a traditional Cox proportional hazard (CoxPH) model in predicting the OS of PNENs in this study. Finally, the best model will be implemented in available software for clinical use.

## 2 METHOD

### 2.1 Data collection

We retrospectively collected and analyzed data of patients with PNENs from the SEER database (Version 8.4.0; National Cancer Institute, Bethesda, MD) from the 18 cancer registries from 2000 to 2018. SEER data are open to the public, and approval from the local ethics committee is not needed.

The following variables from SEER were obtained: age, sex, marital status, race, primary tumor site, AJCC TNM stage (seventh edition), grade, treatment methods (surgical type, radiotherapy, and chemotherapy), tumor size, tumor number, tumor extension, distant metastasis, survival months and status. In the pathological grading system, PNENs were divided into a well‐differentiated group and a poorly differentiated group.

Patient data were retrieved based on the 3rd edition International Classification of Diseases for Oncology (ICD‐O‐3) and the primary site code for the pancreas (C25.0–C25.9). The following histology diagnosis codes were included: islet‐cell adenocarcinoma (8150), malignant beta‐cell tumor (8151), malignant alpha‐cell tumor (8152), G‐cell tumor (8153), VIPoma (8155), malignant somatostatinoma (8156), carcinoid tumor (8240), enterochromaffin cell tumor (8242), mucocarcinoid tumor (8243), neuroendocrine carcinoid (8246), and atypical carcinoid tumor (8249). Exclusion criteria were as follows: patients without survival time or survival time of less than 1 month, PNEN was not affirmed as the primary tumor site, and unknown TNM stage. Finally, a total of 3239 PNEN patients were enrolled for subsequent analysis in this study.

### 2.2 Feature selection

Correlations between clinicopathological features were calculated using the STATS R package. Highly collinear variables with Pearson correlation values greater than 0.7 were excluded because they would overfit the model.^11^ The prognosis of the characteristic was evaluated using univariate and multivariate Cox regression methods. The final features obtained from the above analysis methods were used for the development of the models.

### 2.3 Data preprocessing

We preprocessed the data extracted from the SEER database. Unordered categorical variables were quantified and converted into binary categorical variables using the one‐hot encoding method. Continuous variables did not require any special treatment. The MissForest method was used to interpolate the missing data, which was suitable for continuous and categorical variables. Its core algorithm was to use known variables as independent variables and those with missing values as dependent variables to establish a random forest to predict missing values.^12^

### 2.4 Model development

In this study, the patients from the SEER database were divided into a training set and a testing set at a ratio of 7:3. Four popular algorithms, including DeepSurv, the neural multitask logistic model (NMLTR), random survival forest (RSF), and the standard CoxPH model, were selected to develop the training model. CoxPH is a semiparametric regression model that analyzes the impact of multiple variables on patient survival. RSF is a combination of random forest and survival analysis methods that can assess the risk function and analyze right‐censored data by using integrated prediction of multiple decision trees.^13^ As the prognostic neural network model based on Cox regression analysis, DeepSurv is more stable than linear regression or random survival forest prediction.^14^ NMLTR is based on multitask logistic regression, which can improve the network structure and loss function. Moreover, on the training dataset, fivefold cross validation was used to adjust the hyperparameters, and then the parameter combination with the best predictive ability was selected.

### 2.5 Model performance evaluation

After obtaining the final models, the accuracy and overall prediction performance of each model on the testing and training datasets were evaluated and compared using the concordance index (*C*‐index)^15^ and integrated Brier score (IBS).^16^ The sensitivity and specificity of the prediction models were evaluated using the receiver operating characteristic (ROC) curve and the area under the curve (AUC). The clinical usefulness and net benefit of the models were assessed using decision curve analysis (DCA).^17^

### 2.6 Model importance

First, we permuted the data of the features in the testing dataset to calculate the importance of the clinicopathologic characteristics. The accuracy of the model was then calculated by using the displacement data to determine the feature importance.^18^

### 2.7 Algorithm deployment

The “Streamlit” package in Python was used to develop an online application of PNEN survival probability based on the model with the best performance.

### 2.8 Statistical analysis

Continuous variables such as clinical and pathological features are displayed as the mean value ± standard deviation. Frequencies (*n*) and percentages (%) were used to represent categorical variables. For statistical analysis, we used the chi‐square test and Student's *t* test. Python (version 3.3.8) was applied to construct the ML models. The date of initial diagnosis to death or last follow‐up was used to represent OS. All calculations and analyses were performed in R software (version 4.2.1; the R Foundation for Statistical Computing). *p* < 0.05 was considered statistically significant in this study.

## 3 RESULTS

### 3.1 Patient characteristics

The study collected data from a total of 3239 patients diagnosed with PNENs in the SEER database from 2000 to 2018, and Table 1 presents the clinicopathological features of these patients. There were 1451 (45.0%) females and 1788 (55.0%) males. The median age at diagnosis was 60 years (range: 50–69 years). A total of 38.0% of patients were not married, and 62.0% were married. Seventy‐nine percent of patients were white, 12% were black, and 8.9% were other. According to the primary site of PNENs, 1032 (32%) were in the head of the pancreas, 963 (30%) were in the tail, 420 (13%) were in the body, and 833 (26%) were in other sites. There were 814 (25%) patients in stage I, 603 (19%) in stage II, 103 (3.2%) in stage III, and 1719 (53%) in stage IV. The grading results showed that 81.2% of the tumors were well differentiated, and 18.8% were poorly differentiated. For the surgical method, 1744 (55%) patients did not receive surgery, 1278 (40%) received local or partial pancreatectomy, and 169 (5.3%) received total or extended pancreatectomy. In addition, 94 (2.9%) patients received radiotherapy, and 1093 (34%) patients received chemotherapy. For the tumor size, the median size was 38 mm (range: 24–60 mm). For the tumor number, 2965 (92%) cases had one tumor, and 274 (8.5%) had more than one tumor. Distant metastases occurred in 1719 (53%) patients. Localized tumors accounted for 56%, no vascular invasion accounted for 32%, and further extension accounted for 12%. The median OS was 49 (13–28) months, and 57% of patients died.

**TABLE 1:** Clinicopathological characteristic and Cox regression analysis of patients with PNENs.

Characteristic	Overall	Unicox			Multicox		
	*N* = 3239^a^	HR	95% CI	*p*‐value	HR	95% CI	*p*‐value
Age	60 (50, 69)	1.02	1.02, 1.03	**<0.001**	1.03	1.02, 1.03	**<0.001**
Gender							
Female	1451 (45%)	‐	‐		‐	‐	
Male	1788 (55%)	1.12	1.02, 1.23	**0.015**	1.29	1.09, 1.52	**0.003**
Marital status							
Not married	1235 (38%)	‐	‐		‐	‐	
Married	2004 (62%)	0.81	0.74, 0.89	**<0.001**	0.86	0.73, 1.02	0.078
Race							
White	2554 (79%)	‐	‐		‐	‐	
Black	393 (12%)	1.11	0.97, 1.27	0.14	1.18	0.92, 1.51	0.19
Other	287 (8.9%)	0.81	0.68, 0.96	**0.016**	0.87	0.65, 1.16	0.34
Unknown	5						
Primary site							
Head	1023 (32%)	‐	‐		‐	‐	
Body	420 (13%)	0.81	0.70, 0.95	**0.009**	0.81	0.61, 1.06	0.12
Tail	963 (30%)	0.80	0.71, 0.90	**<0.001**	0.80	0.65, 0.97	**0.022**
Other	833 (26%)	1.18	1.05, 1.33	**0.004**	0.78	0.63, 0.98	**0.030**
Stage							
I	814 (25%)	‐	‐		‐	‐	
II	603 (19%)	2.07	1.70, 2.54	**<0.001**	1.55	1.15, 2.10	**0.004**
III	103 (3.2%)	4.51	3.38, 6.02	**<0.001**	2.02	1.19, 3.42	**0.009**
IV	1719 (53%)	7.89	6.69, 9.30	**<0.001**	3.45	2.61, 4.55	**<0.001**
Grade							
Well differentiated	1482 (81.2%)	‐	‐		‐	‐	
Poorly differentiated	343 (18.8%)	6.55	5.23, 7.65	**<0.001**	3.68	2.72, 4.66	**<0.001**
Unknown	1414						
Surgery							
None	1744 (55%)	‐	‐		‐	‐	
Local or partial pancreatectomy	1278 (40%)	0.17	0.15, 0.20	**<0.001**	0.35	0.28, 0.43	**<0.001**
Total or extended pancreatectomy	169 (5.3%)	0.22	0.17, 0.28	**<0.001**	0.38	0.27, 0.54	**<0.001**
Unknown	48						
Radiotherapy	94 (2.9%)						
No		‐	‐		‐	‐	
Yes		1.18	0.92, 1.52	0.19	1.24	0.88, 1.73	0.22
Chemotherapy	1093 (34%)						
No		‐	‐		‐	‐	
Yes		2.71	2.47, 2.97	**<0.001**	1.14	0.93, 1.40	0.21
Tumor size, mm	38 (24, 60)	1.00	1.00, 1.00	**<0.001**	1.00	1.00, 1.00	0.41
Unknown	446						
Number of tumors							
1	2965 (92%)	‐	‐		‐	‐	
>1	274 (8.5%)	0.62	0.52, 0.75	**<0.001**	1.18	0.93, 1.50	0.18
Tumor extension							
Localized	1562 (56%)	‐	‐		‐	‐	
No vascular invasion	909 (32%)	1.45	1.30, 1.63	**<0.001**	1.20	0.98, 1.47	0.082
Further extension	335 (12%)	2.33	2.01, 2.69	**<0.001**	0.80	0.59, 1.08	0.14
Unknown	433						
Distant metastasis							
Not	1516 (47%)	‐	‐		‐	‐	
Yes	1719 (53%)	4.94	4.44, 5.49	**<0.001**			
Unknown	4						
Survival months	49 (13, 82)						
Status							
Alive	1378 (43%)						
Dead	1861 (57%)						

*Note*: Bold indicates *p* value less than 0.05.

Abbreviations: CI, confidence interval; HR, hazard ratio; IQR, interquartile range; PNEN, pancreatic neuroendocrine neoplasm.

^a^ Median (IQR); *n* (%).

### 3.2 Feature selection and data preprocessing

**FIGURE 1:**
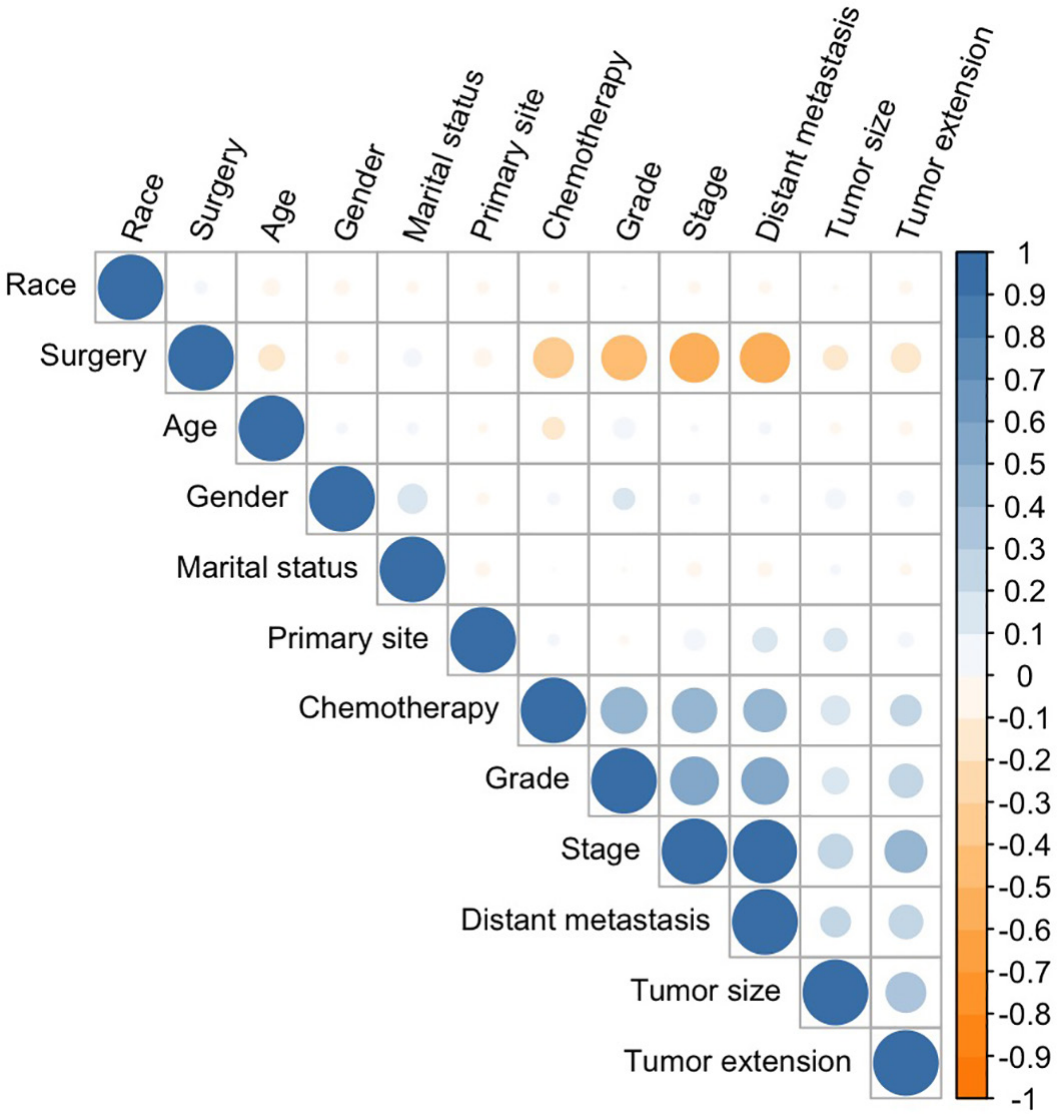
Correlation coefficients for variables in the dataset. The value of the correlation coefficient varied between +1 and 1. Blue represents a positive correlation, and yellow represents a negative correlation.

The univariate Cox regression analysis revealed that most clinicopathological characteristics except for race and radiotherapy were significantly associated with OS in patients with PNENs (Table 1). According to the results of the multivariate Cox regression, the independent prognostic factors for OS were age (*p* < 0.001), sex (*p* < 0.01), stage (*p* < 0.01) and surgery (*p* < 0.001). There was a high degree of collinearity between stage and distant metastasis, as shown in Figure 1. Overall, we finally included the following 10 characteristics in the model development: age, sex, marital status, race, primary tumor site, grade, surgery, chemotherapy, tumor size, and tumor extension. Next, according to a ratio of 7:3, we divided the dataset into a training set (2268 cases) and a testing set (971 cases). The data distribution of these features is shown in Table 2.

**TABLE 2:** Distribution of characteristic in testing dataset and training dataset.

	Level	Overall	Test	Train	*p*‐value
*n*		3239	971	2268	
Age [mean (SD)]		59.11 (13.8)	59.08 (13.9)	59.12 (13.8)	0.927
Gender (%)	Female	1451 (44.8)	433 (44.6)	1018 (44.9)	0.909
	Male	1788 (55.2)	538 (55.4)	1250 (55.1)	
Marital status (%)	Not married	1235 (38.1)	386 (39.8)	849 (37.4)	0.228
	Married	2004 (61.9)	585 (60.2)	1419 (62.6)	
Race (%)	White	2554 (79.0)	778 (80.1)	1776 (78.5)	0.370
	Black	393 (12.2)	106 (10.9)	287 (12.7)	
	Other	287 (8.9)	87 (9.0)	200 (8.8)	
Primary site (%)	Head	1023 (31.6)	310 (31.9)	713 (31.4)	0.883
	Body	420 (13.0)	121 (12.5)	299 (13.2)	
	Tail	963 (29.7)	284 (29.2)	679 (29.9)	
	Other	833 (25.7)	256 (26.4)	577 (25.4)	
Grade (%)	Well differentiated	1482 (81.2)	429 (80.8)	1053 (81.4)	0.873
	Poorly differentiated	343 (18.8)	102 (19.2)	241 (18.6)	
Surgery (%)	None	1744 (54.7)	532 (56.0)	1212 (54.1)	0.603
	Local or partial pancreatectomy	1278 (40.1)	370 (38.9)	908 (40.5)	
	Total or extended pancreatectomy	169 (5.3)	48 (5.1)	121 (5.4)	
Chemotherapy (%)	No	2146 (66.3)	631 (65.0)	1515 (66.8)	0.337
	Yes	1093 (33.7)	340 (35.0)	753 (33.2)	
Tumor size [mean (SD)]		46.07 (37.5)	47.45 (44.7)	45.48 (34.0)	0.203
Tumor extension (%)	Localized	1562 (55.7)	450 (53.3)	1112 (56.7)	0.205
	No vascular invasion	909 (32.4)	283 (33.5)	626 (31.9)	
	Further extension	335 (11.9)	111 (13.2)	224 (11.4)	
Survival months [mean (SD)]		53.17 (43.1)	52.54 (42.9)	53.45 (43.2)	0.583
Status (%)	Alive	1378 (42.5)	414 (42.6)	964 (42.5)	0.975
	Dead	1861 (57.5)	557 (57.4)	1304 (57.5)	

### 3.3 Model performance comparisons

**FIGURE 2:**
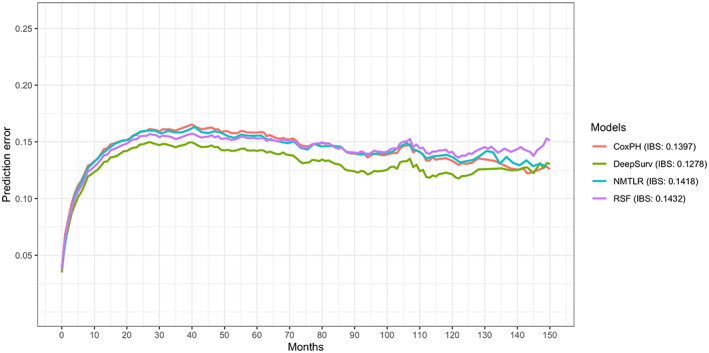
The prediction error curves of the CoxPH, DeepSurv, NMTLR, and RSF models. A lower IBS (usually less than 0.25) indicated better prediction performance. CoxPH, Cox proportional hazard; IBS, integrated Brier score; NMLTR, neural multitask logistic; RSF, random survival forest.

To confirm the best predictive model, several methods were used to compare the performance between the CoxPH, NMLTR, DeepSurv, and RSF models. Table 3 shows the values of the *C*‐index and IBS for these models. In the testing set, the DeepSurv model had the highest *C*‐index value of 0.7882 compared with the CoxPH, NMLTR and RSF models (*C*‐index = 0.7501, 0.7616, and 0.7612, respectively). The IBS of the CoxPH, NMLTR, DeepSurv, and RSF models were 0.1397, 0.1418, 0.1278 and 0.1432, respectively (Table 3; Figure 2). The higher value of the *C*‐index and lower value of the IBS indicated that the DeepSurv model performed better than the other models.

**TABLE 3:** Performance of four survival models.

	*C*‐index		IBS	5‐year AUC	10‐year AUC
	Train^a^	Test^a^			
CoxPH	0.7549	0.7501	0.1397	0.85	0.87
NMLTR	0.7679	0.7616	0.1418	0.85	0.87
DeepSurv	0.7892	0.7882	0.1278	0.87	0.90
RSF	0.7770	0.7612	0.1432	0.85	0.86

^a^ *C*‐index in train and test dataset were calculated separately, the other three metrics were calculated in the testing set.

Abbreviations: AUC, area under the curve; CoxPH, standard cox proportional hazards; IBS, integrated Brier score; NMLTR, neural multitask logistic regression; RSF, random survival forest.

**FIGURE 3:**
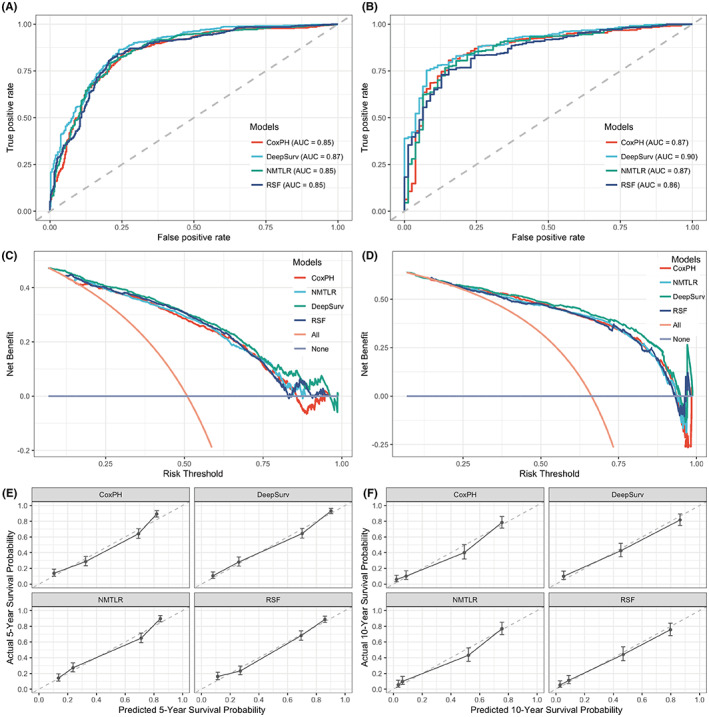
The ROC, DCA and calibration curves for 5‐ and 10‐year survival predictions of CoxPH, DeepSurv, NMTLR, and RSF models. ROC curves for the (A) 5‐ and (B) 10‐year OS predictive models. DCA curves for (C) 5‐ and (D) 10‐year OS. Calibration plots for predicting (E) 5‐ and (F) 10‐year OS. CoxPH, Cox proportional hazard; DCA, decision curve analysis; IBS, integrated Brier score; NMLTR, neural multitask logistic; OS, overall survival; ROC, receiver operating characteristic; RSF, random survival forest.

To assess the accuracy of the four models, ROC curves were constructed. The results showed that the DeepSurv model had the best predictive performance for 5‐ and 10‐year OS (AUC = 0.87 and 0.90), followed by CoxPH (AUC = 0.85 and 0.87), NMLTR (AUC = 0.85 and 0.87) and RSF (AUC = 0.85 and 0.85) (Figure 3A,B). In addition, we also performed the ROC curve of the seventh AJCC TNM staging system to compare the predictive model with commonly used prognostic classifications and found that the AUCs of the four models in the testing set were all larger than that of the AJCC staging system for both 5‐ and 10‐year OS (AUC = 0.721 and 0.805) (Figure S1). The DCA results showed that all the prognostic models had good positive net benefit at the 5‐ and 10‐year time points (Figure 3C,D). Followed by the NMTLR and CoxPH models, the calibration plots of 5‐ and 10‐year OS suggested the best concordance between the predictive values and observed probabilities in the DeepSurv and RSF models (Figure 3E,F). The above results suggested that the DeepSurv model had higher performance and accuracy than the NMLTR, RSF and classical CoxPH models in predicting the survival prognosis of patients with PNENs.

### 3.4 Performance of the models tested by risk stratification of survival time

**FIGURE 4:**
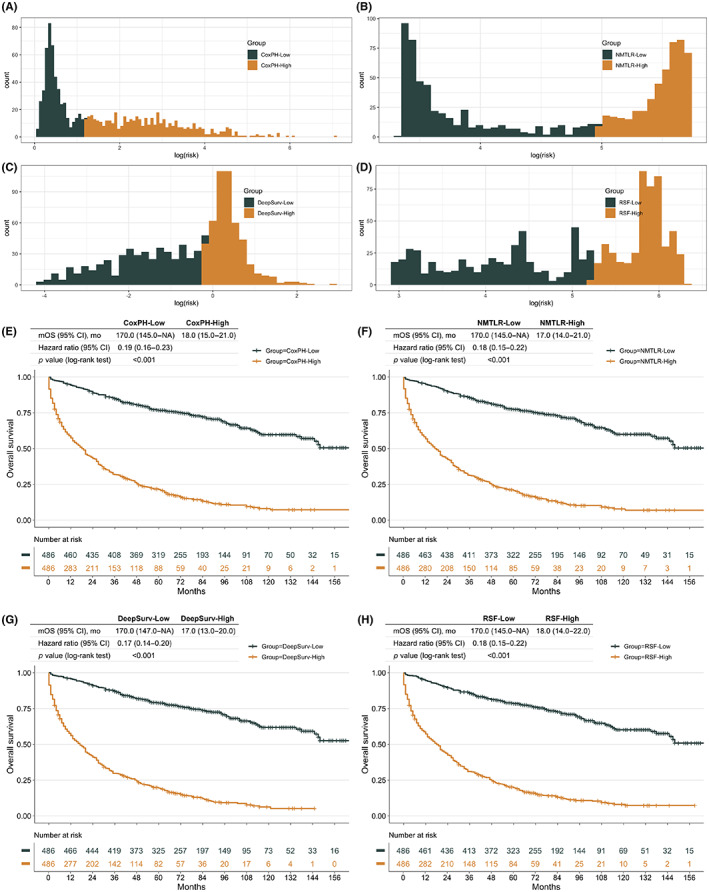
Test performance of DeepSurv, NMLTR and RSF models. Patients were divided into high‐risk and low‐risk groups according to the median risk score of the (A) CoxPH model, (B) NMTLR model, (C) DeepSurv model, and (D) RSF model. Kaplan–Meier analysis of OS for the (E) CoxPH model's risk scores, (F) NMTLR model's risk scores, (G) DeepSurv model's risk scores, and (H) RSF model's risk scores. CoxPH, Cox proportional hazard; IBS, integrated Brier score; NMLTR, neural multitask logistic; OS, overall survival; RSF, random survival forest.

Patients with PNENs were classified into high‐ and low‐risk groups based on median risk scores of the CoxPH, NMLTR, DeepSurv, and RSF models (Figure 4A–D). The OS of patients in the high‐risk group was shorter than that of patients in the low‐risk group in the four models (*p* < 0.001, Figure 4E–H). For stratifying the survival probability of PNEN patients, the findings revealed that the four models all showed good performance.

### 3.5 Feature importance

**FIGURE 5:**
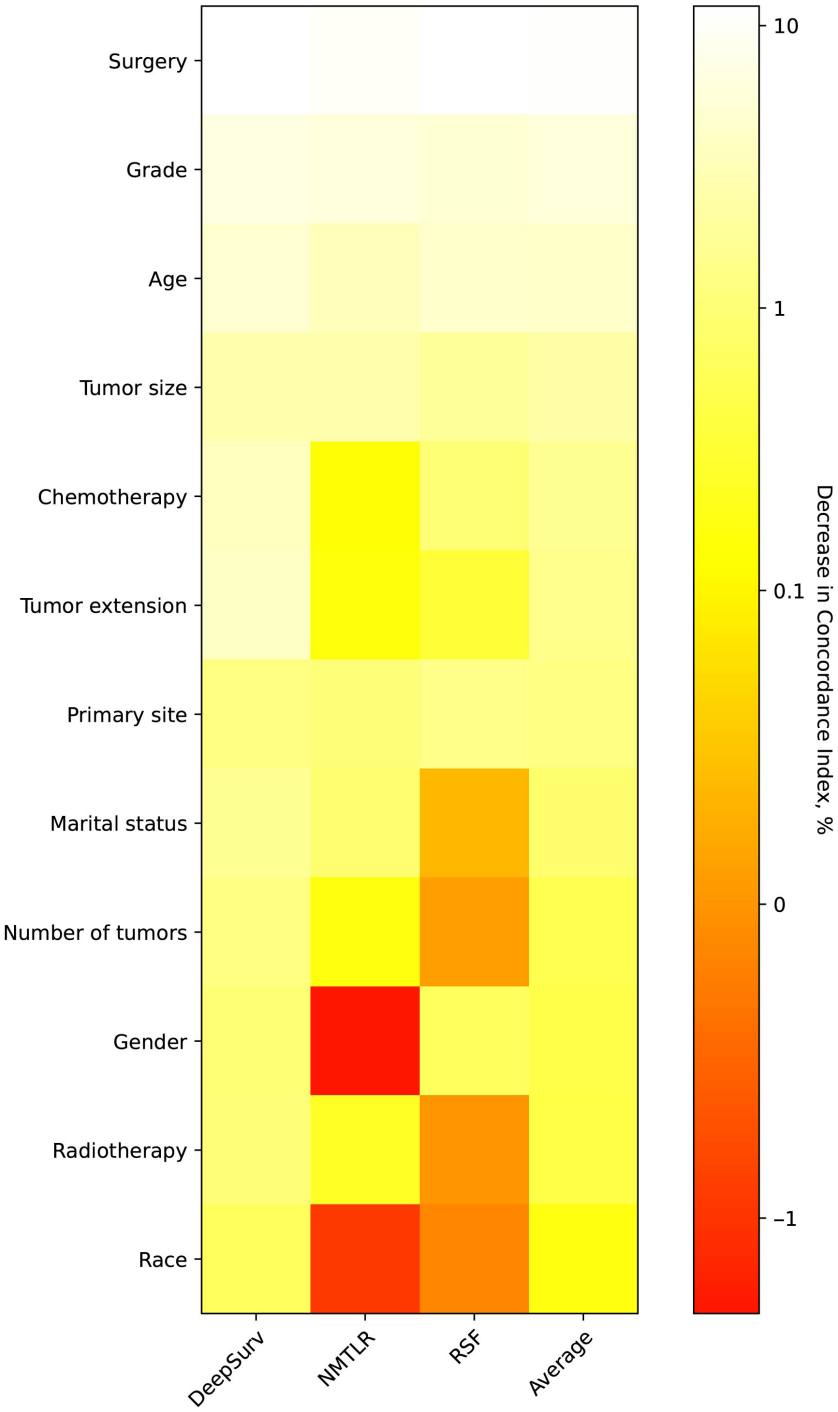
Heatmap of feature importance for the DeepSurv, NMLTR and RSF models. The values were expressed as the percentage decrease in the *C*‐index after replacement. The higher the values of the feature suggested that it was more important to the predictive accuracy of the deep learning models. NMLTR, neural multitask logistic; RSF, random survival forest

Figure 5 presents the feature importance for the accuracy of the DeepSurv, NMLTR and RSF prognostic models. The C‐index was reduced on average by more than 1% with surgery, grade, age, chemotherapy, tumor extension, primary tumor site and tumor size.

### 3.6 Development of the online survival application

**FIGURE 6:**
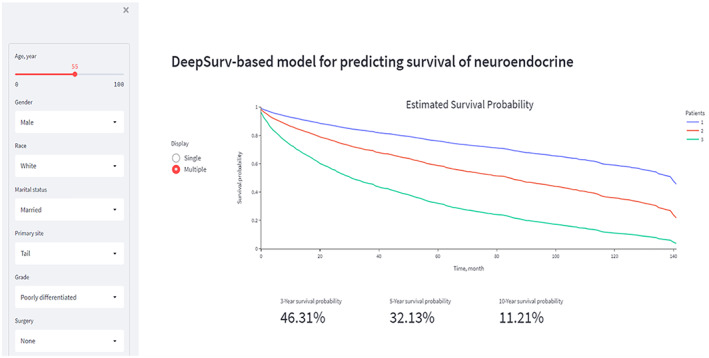
Snapshot of a web‐based application of the DeepSurv model for pancreatic neuroendocrine neoplasm survival probability.

To better apply the conclusions of this study to clinical practice, we developed a web‐based PNEN survival probability application based on the DeepSurv model (https://whuh‐ml‐neuroendocrinetumor‐app‐predict‐oyw5km.streamlit.app/). This website could provide clinicians, patients, and researchers with the 3‐, 5‐ and 10‐year survival probabilities of PNENs. The illustration of the online application is also shown in Figure 6.

## 4 DISCUSSION

In this study, we used the SEER database to collect the important clinicopathological characteristics of PNEN patients and constructed prognostic models of PNENs through three common ML algorithms (NMTLR, RSF, and DeepSurv) and the classical CoxPH model. We first performed CoxPH regression and collinearity analysis to determine variables related to the prognosis of 3239 patients with PNENs. Finally, age, sex, marital status, race, primary site, grade, surgery, chemotherapy, tumor size, and tumor extension were included in the models. A variety of methods were applied to compare the prediction effectiveness of each model for PNENs.

PNEN is a rare heterogeneous tumor that is usually diagnosed at an advanced stage and has a poor prognosis.^19^ Early detection and treatment are crucial to improve the prognosis of these patients. At present, there are several prognostic detection systems to assess the prognosis of patients with PNENs. Zhai et al. found that based on the SEER database, grading was superior to TNM staging in predicting the survival prognosis of PNENs.^20^ A meta‐analysis by Gao et al. showed that surgical margin, G‐stage, TNM stage, lymph node, metastasis, vascular invasion and necrosis were associated with the prognosis of PNENs.^21^ In addition, one study established a nomogram of recurrence after radical resection of PNENs and found that the number of positive lymph nodes, tumor diameter, Ki‐67 index and perineural or vascular invasion were prognostic factors.^22^ In addition to these factors, primary tumor site, grade, surgical procedure, chemotherapy, race, and marital status were included in the model of this study.

In recent years, ML has been widely applied in medical imaging, auxiliary diagnosis and disease prediction of NENs. Klimov et al. established a novel ML method to predict metastatic risk in PNENs.^23^ The pathological grade of patients with PNENs might be identified precisely using CT combined with ML.^24^, ^25^ However, ML has not yet been reported in predicting the survival of PNENs. Therefore, this study aimed to focus on the role of ML models in the prognosis of PNENs. We established NMTLR, RSF, DeepSurv and CoxPH models and then evaluated the performance of these four models with the *C*‐index, IBS, ROC, calibration chart and DCA. Our study showed that compared with the CoxPH model, the *C*‐index of the NMTLR, RSF and DeepSurv models was higher in the training set and testing set, indicating that the prediction performance of ML was better than that of CoxPH regression. Among the three ML models, the DeepSurv model had the best prognostic performance. Moreover, the IBS of the DeepSurv model was the lowest, and the AUC area was the largest in predicting the 5‐ and 10‐year OS. Additionally, we also found that the AUCs of the four models to predict 5‐ and 10‐year OS were all larger than those of the traditional AJCC staging system. All of these findings suggested that the DeepSurv model was more accurate in predicting the survival of patients with PNENs.

Previous studies often used Cox regression to assess the survival of PNEN patients, which can evaluate the effects of multiple factors on survival time simultaneously but cannot identify complex nonlinear relationships among variables.^26^ In contrast, ML can incorporate nonlinear factors well into the impact on the results. As a prognostic neural network model, DeepSurv had more stable prediction ability than linear regression or RSF.^14^ Oei et al. found that the conditional survival forest and DeepSurv model were better than the CoxPH model in predicting the survival prognosis of nasopharyngeal carcinoma with the C‐index and IBS.^27^Kim et al. found that the DeepSurv model was more precise in predicting the survival rate of oral cancer patients than the RSF and Cox proportional hazards models.^28^ Yan et al. found that DeepSurv was the most successful model in predicting the prognosis of chondrosarcoma compared with the RSF, CoxPH, and NMTLR models.^11^ Deep learning can convert linear and nonlinear predictor variables into linear combinations to predict the prognosis of patients by using multistage neural networks and reduce the structural bias associated with missing follow‐up information, which can predict the survival probability of patients at any time point.^27^, ^29^ Therefore, when dealing with large samples and multivariate and nonlinear data, the DeepSurv model has obvious advantages in prediction compared with other models.

Finally, to provide clinicians, patients and researchers with more convenient data visualization, we developed a free online application based on the DeepSurv algorithm in this study. The application can dynamically predict the OS probability of PNEN patients with different clinicopathological features at different time points, which can be accessed via the website https://whuh‐ml‐neuroendocrinetumor‐app‐predict‐oyw5km.streamlit.app/.

However, there were some limitations in our study. First, this study only included data from patients with PNENs in some regions of the United States, and we did not verify the established predictive model in the external dataset. The clinicopathological data of PNEN patients from other centers are not easy to obtain due to the particularity of clinicopathological information. In addition, we could only access the inferred data of previously published articles, rather than the original data, which cannot be verified externally. We will collect relevant clinicopathological information from PNEN patients to further evaluate the reliability of the DeepSurv method in future studies. Second, the specific chemotherapy methods, which are significant predictive factors for PNENs, are not included in the SEER database. Third, prospective studies are required to provide more reliable evidence of the predictive performance of the models.

## 5 CONCLUSION

In conclusion, based on the basic clinical characteristics of PNENs in the SEER database, this study established and compared three ML algorithms and a traditional algorithm to predict the survival performance of patients with PNENs. Studies have shown that all four models could predict the prognosis of PNENs well, among which the DeepSurv model had the best prediction performance. As a deep learning algorithm, the DeepSurv model had accuracy rates of 87% and 90% in predicting the 5‐ and 10‐year OS, respectively, which had a certain potential clinical application value. We finally developed an application based on the DeepSurv model that could provide reliable individual survival information for patients with PNENs and help with clinical decision‐making.

## AUTHOR CONTRIBUTIONS

**Chen Jiang:** Data curation (equal); methodology (equal); writing – original draft (lead); writing – review and editing (equal). **Kan Wang:** Validation (equal); writing – review and editing (equal). **Lizhao Yan:** Resources (equal); writing – review and editing (equal). **Hailing Yao:** Validation (equal). **Huiying Shi:** Methodology (equal); supervision (equal). **Rong Lin:** Supervision (equal); writing – review and editing (equal).

## FUNDING INFORMATION

Supported by the National Natural Science Foundation of China (Nos. 81974068, 82100569, 82170571), the Natural Science Foundation of Hubei Province Youth Project (No. 2021CFB122), the Key project of Hubei Natural Science Foundation (No. 2022CFA009). The funding agency was not involved in the study's planning, the data collection and analysis, or in writing the paper.

## CONFLICT OF INTEREST STATEMENT

There are no competing interests in this work.

## STATEMENT OF ETHICS

The SEER database is open to the public, there is no need for an ethical committee permission before using it in this work.

## Supporting information


Figure S1.

## Data Availability

The data used and/or analyzed in this study were obtained from the SEER database, which can be found as follow: https://seer.cancer.gov.
