# Supplementary figures and images for: Predicting the survival of patients with pancreatic neuroendocrine neoplasms using deep learning: A study based on Surveillance, Epidemiology, and End Results database
Source: Cancer Med. 2023 May 11;12(11):12413–24. doi: 10.1002/cam4.5949 (PMC10278508; doi:10.1002/cam4.5949)

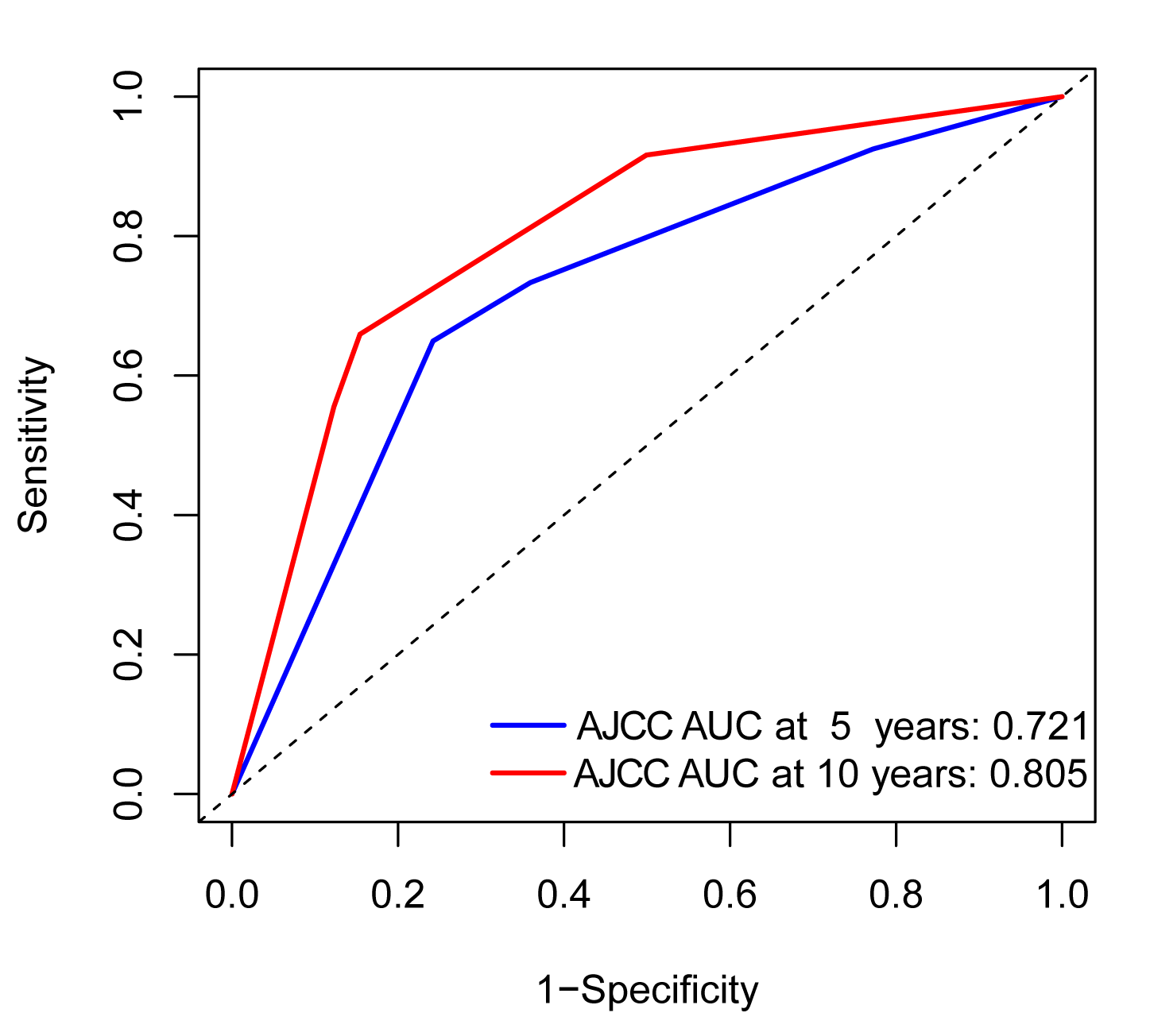

Supplement: Supplementary file 1 — Figure S1. [file CAM4-12-12413-s001.tif]
